# Identification and Molecular Characterization of a Chitin Deacetylase from *Bombyx mori* Peritrophic Membrane

**DOI:** 10.3390/ijms15021946

**Published:** 2014-01-27

**Authors:** Xiao-Wu Zhong, Xiao-Huan Wang, Xiang Tan, Qing-You Xia, Zhong-Huai Xiang, Ping Zhao

**Affiliations:** 1 State Key Laboratory of Silkworm Genome Biology, Southwest University, Chongqing 400716, China; E-Mails: zxw_strive@163.com (X.-W.Z.); wangxiaohuan1024@126.com (X.-H.W.); luffy.tan@gmail.com (X.T.); xiaqy@swu.edu.cn (Q.-Y.X.); xbxzh@swu.edu.cn (Z.-H.X.); 2 Translational Medicine Research Center, North Sichuan Medical College, Nanchong 637000, Sichuan, China

**Keywords:** *Bombyx mori*, peritrophic membrane, 2-D electrophoresis, chitin deacetylase, phylogenetic relationship

## Abstract

The insect midgut epithelium is generally lined with a unique chitin and protein structure, the peritrophic membrane (PM), which facilitates food digestion and protects the gut epithelium. PM proteins are important determinants for PM structure and formation. In this study, the silkworm *Bombyx mori* midgut PM protein *BmCDA7* was identified by proteomic tools. The full-length *BmCDA7* cDNA is 1357 bp; the deduced protein is composed of 379 amino acid residues and includes a 16 amino acid residue signal peptide, a putative polysaccharide deacetylase-like domain and 15 cysteine residues present in three clusters. The heterologously expressed proteins of the *BmCDA7* gene in yeast displayed chitin deacetylase activity. Expression of *B. mori BmCDA7* was detected in the midgut at both the transcriptional and translational levels. The *BmCDA7* gene was expressed by the newly hatched silkworm larvae until day seven of the fifth instar and was expressed at a high level in the newly exuviated larvae of different instars. The functions and regulatory mechanism of BmCDA7, however, need further investigation.

## 1. Introduction

The peritrophic membrane (PM), an acellular structure secreted by midgut cells lines the digestive tract in most insects and has important roles in facilitating food digestion and providing protection to the gut epithelium. The PM is composed mainly of proteins, glycosaminoglycans and chitins [1], but only ~30 PM proteins have been isolated and characterized from a variety of insect species. Characterization of structural peritrophic matrix proteins has focused mainly on the following classes: peritrophins; invertebrate intestinal mucins; and proteins with chitin deacetylase domains [2,3]. The peritrophins have the conserved consensus motif CX_13−20_CX_5−6_CX_9−19_CX_10−14_CX_4−14_C peritrophin-A, and this multiple cysteine-rich domain enables the protein to bind to chitin, maintaining the PM structure [4]. Ag-Per1 with two tandem chitin-binding domains (CBDs) is suggested to draw together PM chitin fibrils into a 3-D network [5]. Cb-peritrophin-15 has been identified with only one CBD and might be required to bind to the ends of chitin fibrils [6]. Invertebrate intestinal mucin (IIM) is a highly glycosylated mucin-like protein containing CBDs bound very strongly to the PM. Wang and Granados identified an IIM from the lepidopteran *Trichoplusia ni*, which exhibits a strong association with chitin and is a target substrate for baculovirus enhancement, but is highly resistant to proteolytic attack from endogenous midgut proteases [7]. Chitin deacetylase (CDA; EC 3.5.1.41) is a hydrolytic enzyme that catalyses the hydrolysis of the acetamido group in the *N*-acetylglucosamine units of chitin and chitosan, thus generating glucosamine units and acetic acid [8]. The CDAs were found recently as a new component of insect PM. The first CDA to be purified and characterized was extracted from the fungus *Mucor rouxii* [9]. Studies of CDAs in a variety of species have helped us to understand CDA functions.

CDAs with different biological functions have been found in fungi, yeast and insects. Fungal CDA has an important role in fungal growth, being involved in formation of the fungal cell wall. Davis & Bartnicki-Garcia suggested effective chitosan synthesis requires chitin deacetylase in combination with chitin synthetases operating in tandem in *M. rouxii* [10]. Alfonso *et al.* found a chitin deacetylase activity in *Aspergillus nidulans* that could be implicated in the chitin oligosaccharides during autolysis after the action of endochitinase on cell walls [11]. Another biological role of CDA from the plant pathogen *Colletotrichum lindemuthianum* could modify chitin and allow hyphae to penetrate into plants and act as elicitors for the plant’s defense mechanisms [12,13].

Studies of CDAs in insects are still focused on elucidating the potential biological role of CDA activity and studying the enzymatic process for deacetylation of chitin substrates in the initial stages. Several insect CDA genes have been identified especially from the peritrophic midgut matrix, but the biochemical characteristics of insect CDAs have not been determined [14]. TnPM-P42, a novel midgut PM protein with a CDA-like domain, was first identified from a *T. ni* midgut cDNA expression library. This protein exhibited a strong chitin-binding activity and was associated strongly with the PM [15]. Luschnig *et al.* [16] and Wang *et al.* [17] reported that two *Drosophila melanogaster* CDAs (serpentine and vermiform) affect the synthesis or structure of chitin and showed that they have critical roles in shaping the tracheal tubes as well as regulating the structural properties of the epidermal cuticle. Nine genes encoding CDA-like proteins were presented and expressed in *Tribolium castaneum*, with differing developmental and tissue-specific patterns of expression, suggesting these proteins might have different functions. Results from RNA interference studies showed *TcCDA1* and *TcCDA2* are crucial for insect development and alternatively spliced variants of *TcCDA2*, *TcCDA2A* and *TcCDA2B* have different roles in determining adult cuticle morphology [18]. A downregulated chitin deacetylase-like protein was selected from a group of *Helicoverpa armigera* genes with changed expression levels after infection with *H. armigera* single nucleopolyhedrovirus, which might reduce the susceptibility of this bollworm to baculovirus by decreasing its PM permeability [19]. To date, however, there is only one published report demonstrating chitin deacetylase activity of a *Mamestra configurata* CDA protein, which was examined in inclusion body fractions from *Escherichia coli* Rosetta 2 (DE3) cells expressing recombinant McCDA1 [20].

The silkworm *Bombyx mori* is used as a model for Lepidoptera in sericulture and biotechnology because it is quite large, has a relatively short and predictable life cycle, is highly fertile and adapts readily to laboratory culture [21]. *B. mori* PM has received little study, however, and hence very limited information is available leading to a poor understanding of its biological function. In this study, we used a proteomics approach to examine the proteins associated with the silkworm PM. Here, we report the molecular cloning and sequencing of a cDNA encoding a CDA from *B. mori* PM.

## 2. Results and Discussion

### 2.1. Proteomic Analysis of *B. mori* PM

**Figure 1.**
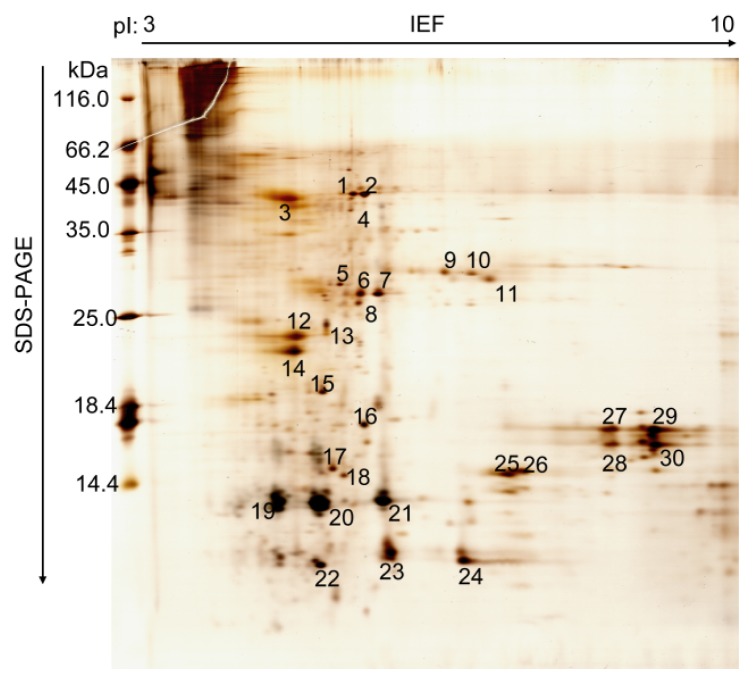
Two-dimensional gel electrophoresis of proteins associated with the peritrophic membrane of *B. mori* larvae. One hundred and fifty micrograms of protein was applied to the IPG strip (18 cm, pH 3–10, L) and 15% SDS-PAGE was carried out for separation in the second dimension. The major expressed protein spots on the gel are numbered.

Until now, the mode of action and catalytic mechanism of CDAs were not well understood. The function of fungal and bacterial CDAs has been demonstrated, including modification of the insect cuticular chitin to aid mycelial penetration and evasion of lysozyme action [8,14]. The role of insect CDAs, however, has had no more detailed investigation. Recently, several CDA genes were identified in the PM of various insects. In this study, we characterized silkworm CDA associated with the PM using a coupled proteomics and genomics approach. To study the protein profiles of silkworm PM, the total proteins were extracted from the silkworm larvae PM on day three of the fifth instar, separated by 2-D polyacrylamide electrophoresis and >60 protein spots were observed (Figure 1). We detected more spots in silkworm PM compared to other insects [20,22–24]. Most of the resolved protein spots had pI values between 5 and 9 and a molecular mass of 10–66 kDa. A total of 30 protein spots from PM were excised from the gel and investigated further by matrix-assisted lased desorption/ionization-time of flight mass spectrometry (MALDI-TOF MS) or liquid chromatography tandem mass spectrometry (LC-MS/MS). In addition, 12 proteins were identified (Table 1) and the detailed MS analysis for the identified silkworm PM proteins is given in Table S1. This investigation revealed these proteins were components of the PM. There were two structural peritrophic matrix proteins, chitin deacetylase (Spot 4) and PM chitin-binding protein 2 (Spot 17). They were also characterized by shotgun proteome technology in recent studies [25,26], which suggested that they have significant functional roles in silkworm PM.

Proteome analyses can identify proteins mediating housekeeping, as well as specialized functions of cells and tissues. Peritrophic membrane chitin-binding protein 2 is a chitin-binding protein, and as the chitin-binding domains indicate, adapt and function in the proteinase-rich gut environment, which was shown with CBP1 and CBP2 of *T. ni* [27]. It was reported that CDAs are important enzymes in the regulation of ecdysis physiology, organ formation and anti-pathogen immunity during insect development. Wang *et al.* proposed the subsequent structural modification of chitin by CDAs selectively instructs the termination of tube elongation to the underlying epithelium in *D. melanogaster* [17]. The presence of a downregulated chitin deacetylase-like protein (HaCDA5a) owing to baculovirus infection would lead to less HaCDA5a protein in the PM and thus increase PM stiffness, which is possibly a mechanism to reduce the susceptibility to baculovirus by decreasing the PM permeability [19].

### 2.2. Sequence Analysis of BmCDA7

**Figure 2.**
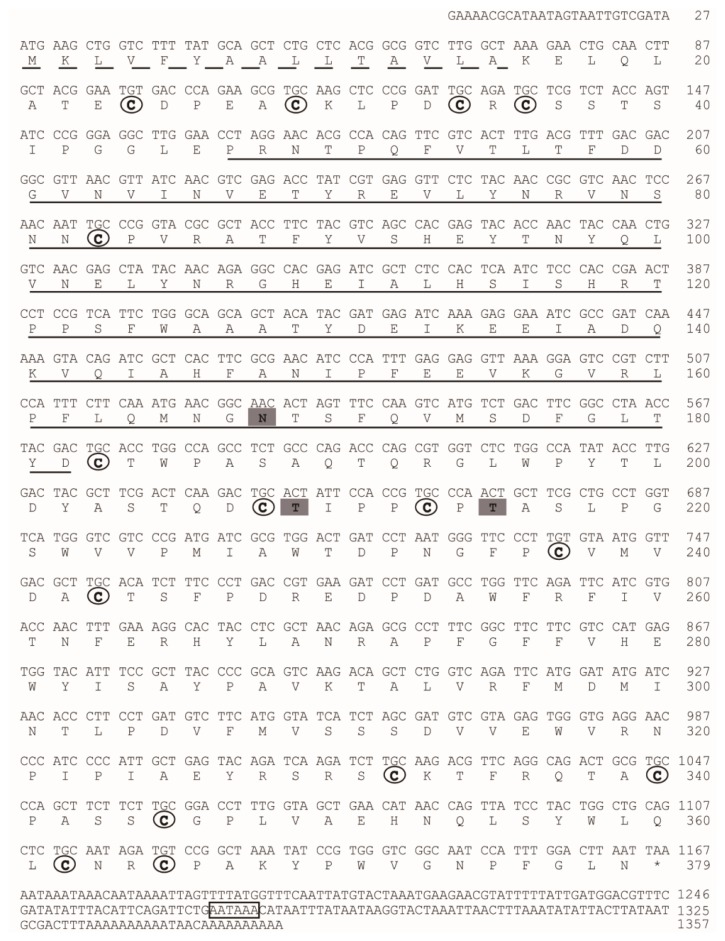
The full-length cDNA sequences and deduced amino acid sequences of BmCDA7. The predicted signal peptide is underlined with a broken line. A potential polyadenylation signal sequence is boxed. The chitin deacetylase domain is underlined. Cysteines in the mature protein sequence are in bold type and circled. The *N*-glycosylation sites and *O*-glycosylation sites are showed with bold type and grey background.

The protein sequence of CDA (BGIBMGA013757) was used to search the silkworm genome database. Another seven genes showing significant homology with CDA were identified (Table S2). We named the silkworm CDA in accord with Dixit *et al.* [28]. BGIBMGA013757, which was identified from PM, was named *BmCDA7*. We cloned and sequenced the *BmCDA7* gene. The cDNA and predicted protein sequences of the *BmCDA7* are shown in Figure 2. Rapid amplification of cDNA ends (RACE) experiments were used to obtain the 5′ and 3′ ends of *BmCDA7*. Finally, the full length of the *BmCDA7* cDNA is 1357 bp followed by an A+T-rich region with two typical polyadenylation signal sequences (AATAAA). The full length of *BmCDA7* cDNA contains an open reading frame of 1140 bp, a 27 bp upstream-untranslated region and a 190 bp downstream-untranslated region. The putative *BmCDA7* cDNA encoded for a 379 amino acid residue protein consisting of a 16 amino acid residue signal peptide according to SignalP software [29] and a mature polypeptide of 363 amino acid residues. After removal of the signal peptide, the deduced protein was predicted by the ExPASy server to have a molecular mass of 41.26 kDa and a theoretical pI of 5.12 [30]. Prediction of potential glycosylation sites using the NetNglyc 1.0 [31] and NetOglyc 3.1 [32] server showed that the protein contains a putative *N*-glycosylation site at Asn168, and putative *O*-glycosylation sites at Thr209 and Thr215. These sequence features suggest that the BmCDA7 protein might be both *N*- and *O*-glycosylated. BmCDA7 has a putative polysaccharide deacetylase-like domain (residues 46–182) and 15 cysteine residues present in three clusters of five situated at residues 24–83, 183–243 and 332–365 as determined by SMART analysis [33]. The polysaccharide deacetylase-like domain showed sequence similarities to the CDA domain sequences from fungi and a bacterium according to Guo *et al.* and the cysteine-rich regions are common to PM proteins. These sequence characteristics are similar to TnPM-P42 of *T. ni*, which is different from the peritrophin-type PM proteins but, instead, has a chitin deacetylase-like domain and uses a CDA-like domain for chitin binding [15]. Therefore, our results indicate BmCDA7 does not resemble any of the peritrophin domains from types I or II PMs [2], but belongs to Class 3 of PM proteins. The distribution patterns of the cysteine residues in BmCDA7 are dissimilar compared to the known peritrophin-type PM proteins. Such arrangements of the cysteine residues could constitute a new type of chitin-binding domain, and provide an important mechanism for the protein-chitin association in PM formation. Wang and Granados suggested the conserved cysteine residues form intra-domain disulfide bonds, which confer the stability of PM proteins in the protease-rich gut environment [34]. They might be a basic module that combines with other protein sequences to generate a new function or modify an existing function.

We downloaded 43 insect CDAs (Table S3) from SilkDB and NCBI to expand the phylogenetic analysis reported by Dixit *et al.* [28]. All of the CDA-like proteins from insects originated from one root. The silkworm CDAs also grouped the proteins into five major groups, I–V, and BmCDA7 was assigned to group V (shown in Figure S1). Dixit *et al.* suggested group V CDA could have a function in insect immunity or alleviate the inhibitory effect of chito-oligosaccharides on the activity of gut chitinases needed for molting [28].

### 2.3. Recombinant Expression and Chitin Deacetylase Activity

**Figure 3.**
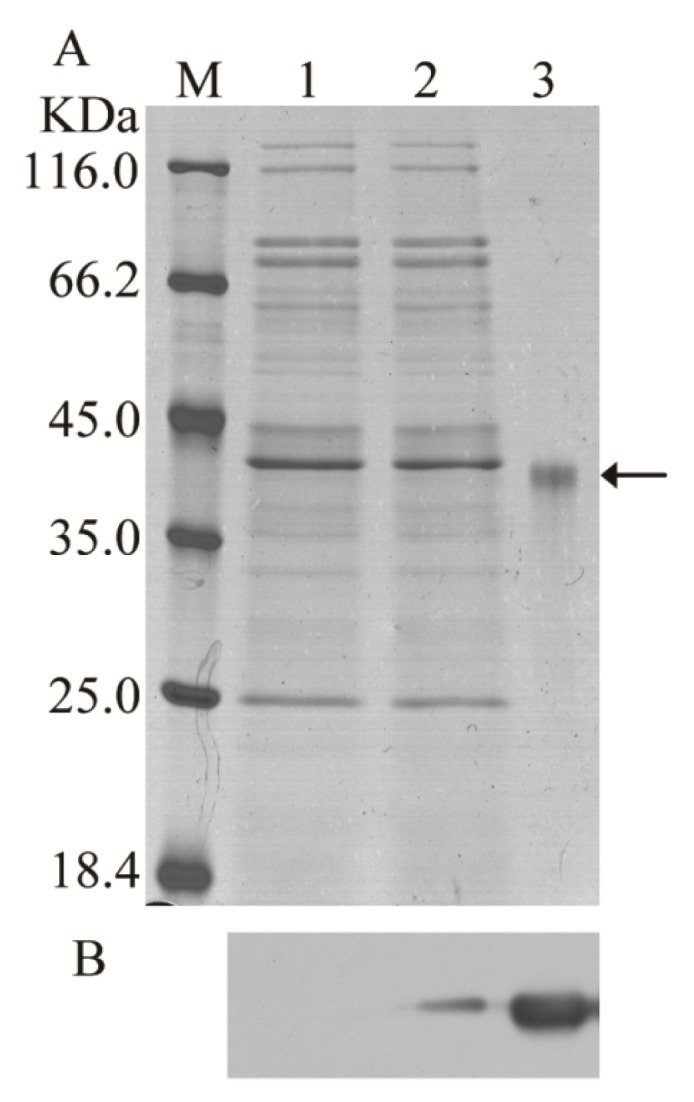
Heterologous expression of the BmCDA7 gene in yeast and bacteria. (**A**) The SDS-PAGE analysis of the heterologous expression for the BmCDA7; (**B**) The western blotting analysis of the heterologous expression for the BmCDA7. Arrow represents the targeted protein. Lane M: Protein marker; Lane 1: The supernatant of the pPIC9K-GS115 induced by 1% methanol for 96 h; Lane 2: The supernatant of the yeast containing recombinant pPIC9K-BmCDA7 plasmid induced by 1% methanol for 96 h; Lane 3: Purified fusion proteins from *E. coli* with His-tag.

*E. coli* BL21 (DE3) was transformed with the recombinant expression vector pET-28a-BmCDA7, and the recombinant protein was expressed abundantly as an insoluble inclusion body after induction with isopropyl β-d-1-thiogalactopyranoside (IPTG) and purified by passage through a Ni^2+^-NTA affinity column (Figure 3A, lane 3). The purified proteins were injected into rabbits to generate polyclonal antibodies. Western blotting was used to determine the specificity of the antibodies. There was one specific band corresponding to the molecular mass of BmCDA7 (41 kDa) as shown in Figure 3A, lane 3. The antibodies were therefore considered suitable for further research.

The recombinant expression vector pPIC9K-BmCDA7 plasmid and the pPIC9K plasmid were transformed into *Pichia pastoris* by electroporation. After transformation and primary screening by histidine-deficient medium and G418, the positive yeast cells containing plasmid were selected for cultivation in Buffered Methanol-Complex Medium (BMMY) after induction by 1% (*v*/*v*) methanol at 30 °C. SDS-PAGE analysis of the crude supernatants at the induction period from 96 h resulted in no significant additional band corresponding to the molecular masses of BmCDA7 in the induced yeast containing recombinant pPIC9K (Figure 3A, the lane 2) but we detected a specific band from crude supernatants of pPIC9K-BmCDA7 using the BmCDA7 antibodies. This result indicated BmCDA7 was expressed in the yeast. Prokaryotic expression of the BmCDA7 protein was present in the inclusion body fraction, and the level of expression was low in yeast. Therefore, we did not purify the recombinant proteins from yeast and used the crude preparation to identify their activities. In earlier studies, CDA was shown to facilitate the entry of fungus into the insect body by converting insect cuticular chitin into chitosan. To date, the CDA activity of recombinant McCDA1 has been examined only in inclusion body fractions from *E. coli* Rosetta 2 (DE3) cells using an in-gel assay [20]. In this study, we used the method as described [35], and found recombinant BmCDA7 was active and the CDA activity of BmCDA7 was 1.85 U/mL (Table 2). Wenling *et al.* reported deacetylation could increase the solubility and decrease the density of chitin fibrils *in vitro* [36]. In other insects, *serpentine* and *vermiform* affected the synthesis or structure of chitin [16,17]. TcCDA2, TcCDA2A and TcCDA2B have different roles in determining adult cuticle morphology [18] and HaCDA5a decreased the PM permeability [19]. We suppose, therefore, that conversion of chitin into chitosan by BmCDA7 might influence the structure and orientation of PM chitin fibrils.

### 2.4. Expression Profiles and Localization of BmCDA7

**Figure 4.**
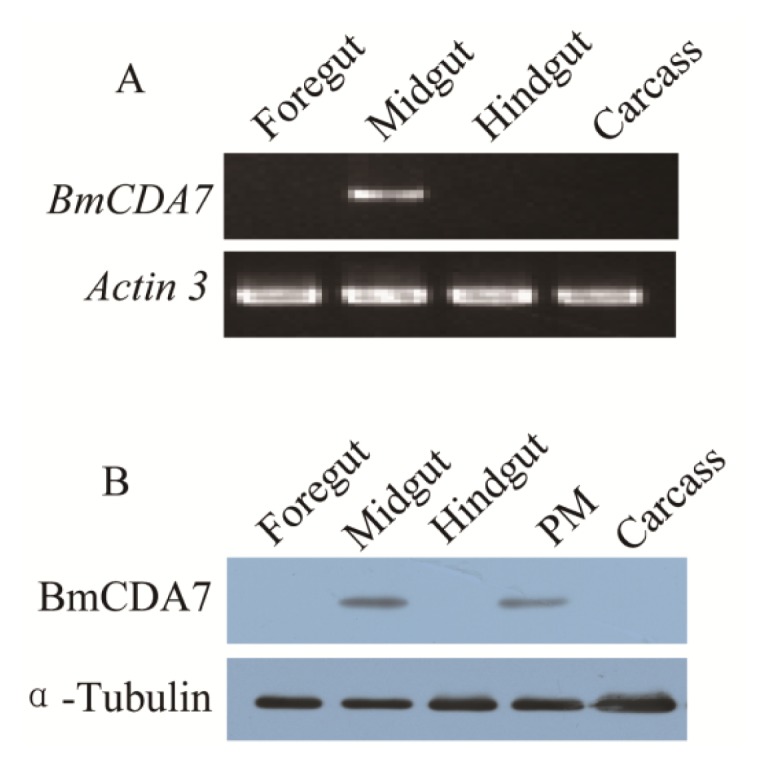
Tissue distribution of BmCDA7. (**A**) The spatial expression profile at the transcriptional level. Total RNA of different tissues from the third day of the fifth instar larvae were used in the RT-PCR analysis. Silkworm actin 3 gene was used as the control; (**B**) Tissue-specific localization of BmCDA7 protein. Western blotting analysis was performed to detect the expression of the protein. Total protein from different tissues from the third day of the fifth instar larvae were used in this analysis. Tubulin was used as the positive control. PM: Peritrophic membrane; Carcass: All tissues minus alimentary canal.

**Figure 5.**
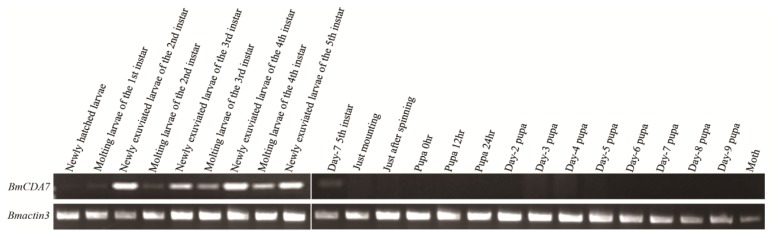
The temporal expression profiles of BmCDA7. To determine the temporal specificity of expression, three whole silkworm bodies (except for the midgut contents) were collected at the times of the first-fourth instar larvae, middle-stage silkworm and molting silkworm. Total RNA was extracted and was used as template for first-strand cDNA synthesis. *Bmactin 3* transcript with the same cDNA template served as an internal control.

The expression of *BmCDA7* was studied by reverse transcription-PCR (RT-PCR; Figure 4A). The foregut, midgut, hindgut and remaining carcass were dissected from larvae on day three of the fifth instar to use in this study. The signal was detected only in the midgut; no signal was found in the foregut, hindgut or remaining carcass. The tissue distribution of BmCDA7 in silkworm larvae was detected by western blot using the antibody to the recombinant protein (Figure 4B). BmCDA7 was detected in the midgut and in PM tissue, but not in the larval foregut, hindgut or remainder of the carcass. This result was confirmed by analysis of the translational levels of *BmCDA7*. This suggests that the BmCDA7 protein is synthesized in the midgut and transferred to the PM, where it is involved in maintaining the PM molecular structure; BmCDA7 is therefore one of the PM proteins. The expression of the *BmCDA7* gene in the newly hatched silkworm larva on day seven of the fifth instar, and a high level of expression in the newly exuviated larvae of different instars, which is the period from formation to the apoptosis of the silkworm midgut, is shown (Figure 5). Therefore, we suggest that the *BmCDA7* gene has important roles in updating the PM. Ghormade *et al.* found the presence of these CDA enzymes in the midgut tissue of larvae only during the feeding period, which might be associated with increased absorption of nutrients [37]. Nahar *et al.* reported CDA had a significant role in self-defense from the insect chitinases produced during the molting process [38].

## 3. Experimental Section

### 3.1. Silkworm Rearing and Sample Preparation

Silkworm strain Dazao obtained from the State Key Laboratory of Silkworm Genome Biology (Southwest University) was chosen for this experiment. Larvae were reared on fresh mulberry (*Morus* sp.) leaves under a 12 h light/12 h dark photoperiod at 26(±1) °C with 75(±5)% relative humidity. The samples were collected at different developmental time points. On day three of the fifth instar, approximately 100 larval PMs were accumulated for two-dimensional polyacrylamide gel electrophoresis (2-DE). Other samples were collected and stored at −80 °C to be used for PCR and western blot experiments. The total PM proteins were extracted in lysis buffer (9 M urea, 4% (*v*/*v*) 3-[(3-cholamidopropyl)dimethylammonio]-1-propanesulfonate (CHAPS), 0.2% (*v*/*v*) Triton X-100, 30 mM dithiothreitol (DTT), 1% (*w*/*v*) Protease Inhibitor Cocktail), homogenized with a tissue grinder, vortex mixed, kept at 4 °C for 1 h and then centrifuged for 30 min at 12,000*g* at 4 °C. Total protein content in the supernatant was determined with a 2-D Quant Kit (GE Healthcare, Milwaukee, MI, USA) as described in the manual.

### 3.2. 2-DE and Image Analysis

Before electrophoresis, 200 μg of total PM proteins was mixed in 350 mL of rehydration buffer I (8.0 M urea, 2% (*v*/*v*) CHAPS, 0.8% (*w*/*v*) DTT, 0.5% IPG buffer, pH 3–10, 0.002% (*w*/*v*) bromophenol blue) and loaded onto an 18 cm broad range IPG strip (pH 3 to 10 NL; GE Healthcare, Milwaukee, MI, USA). Isoelectric focusing (IEF) was done at 20 °C according to the following protocol: 50 V for 12 h, 100 V for 1 h, 200 V for 1 h, 500 V for 30 min, 1000 V for 30 min, 3000 V for 30 min, 5000 V for 30 min then 8000 V until 100,000 Vh. The current limit is 50 μA per IPG strip. Before SDS-PAGE, the IPG strips were equilibrated for 15 min in equilibration buffer I (6 M urea, 50 mM Tris-HCl, pH 8.8, 2% (*w*/*v*) SDS, 30% (*v*/*v*) glycerol, 1% (*w*/*v*) DTT) and later for a further 15 min in equilibration buffer II (equilibration buffer I containing 2.5% (*w*/*v*) iodoacetamide instead of DTT). Equilibrated strips were overlaid onto 15% (*w*/*v*) polyacrylamide gel and subjected to 2-DE in an Ettan DALTsix Electrophoresis System (GE Healthcare, Milwaukee, MI, USA) followed by staining with silver nitrate [39]. At least three biological replicates were performed for PM protein samples. Spots were scanned with a high-resolution image scanner III at 300 pixels and analyzed by ImageMaster 2D software (GE healthcare, Milwaukee, MI, USA).

### 3.3. Protein Digestion and Protein Identification by MS

Thirty major protein spots were cut from the gel by hand and tryptic digestion was done as described [40]. Protein spots were excised and destained with 50 μL of 30 mM potassium ferrocyanide and 50 μL of 100 mM sodium thiosulfate. The pieces of gel were washed twice with 100 μL of Milli-Q-prepared water and dehydrated with 100 μL of acetonitrile (ACN). Next, 10 μL of sequence-grade modified bovine trypsin (10 μg/mL in 25 mM ammonium carbonate; Sigma, St. Louis, MO, USA) was added and incubated overnight at 37 °C. The tryptic peptides were extracted twice by the addition of 50 mM ammonium bicarbonate solution containing 5% TFA and 50% ACN, and concentrated to ~3 μL by vacuum centrifugation (LABCONCO, Kansas City, MO, USA). The tryptic peptides were mixed with equal amounts of α-cyano-4-hydroxycinnamic acid (Sigma, St. Louis, MO, USA) and placed onto sample plates.

Thirteen protein spots were identified using MALDI-TOF MS on a Voyager DE PRO MALDI-TOF MS (Applied Biosystems, Framingham, MA, USA) using delayed ion extraction and positive ion reflectron mode with an accelerating voltage of 20 kV, 60%–65% grid voltage and a delay time of 100 ns. The autolytic peaks of trypsin were used for internal calibration. Mass spectral analysis and protein identification were done as described [41]. The peptide mass fingerprinting (PMF) processed with Data Explorer software was searched against the local database, which was constructed with 23,017 protein sequences (8394 sequences from NCBI and 14,623 sequences from SilkDB) by General Protein/Mass Analysis for Windows software (GPMAW, version 6.10, Lighthouse Data, Odense M, Denmark). MASCOT was used to validate the reliability of the search results.

Four additional protein spots were identified using a Finnigan LTQ mass spectrometer (Thermo, Vernon Hills, IL, USA) as described [26]. The peptide mixtures were separated by reversed-phase HPLC and eluted using a linear gradient from 98% buffer A (0.1% (*v*/*v*) methanoic acid in water) to 80% buffer B (84% ACN, 0.1% (*v*/*v*) methanoic acid in water) at a flow rate of 250 nL/min. Followed by tandem MS analysis, the linear trap quadrupole (LTQ) mass spectrometer was used for peptide detection with the following parameters: source temperature 170 °C, spray voltage 3.0 kV, full scan *m*/*z* range 400–1800. The dynamic exclusion settings were: repeat count 2, repeat duration 0.5 min, exclusion duration 2 min.

### 3.4. Rapid Amplification of cDNA 5′ End (5′ RACE) and 3′ RACE

5′ and 3′ RACE analyses were performed using the GeneRacer kit (Invitrogen, Carlsbad, CA, USA) according to the manufacturer’s instruction. Total RNA was extracted from three midguts of day three of the fifth instar silkworm larvae using Trizol reagent (Invitrogen, Carlsbad, CA, USA), followed by treatment of RNase-free DNase I (Promega, Fitchburg, WI, USA) for 30 min at 37 °C to eliminate the contaminating genomic DNA. The purity of extracted RNA was determined by UV spectrophotometer. Four μg of RNA was reverse-transcribed to the first strand of cDNA using M-MLV reverse transcriptase (Invitrogen, Carlsbad, CA, USA) for 1 h at 42 °C. In order to obtain the full-length cDNA of the BmCDA7, specific primers were designed by primer 5 (Table S4), and then the procedure was performed using the GeneRacer Kit (Invitrogen, Carlsbad, CA, USA) according to the manufacturer’s instructions. All the PCR products were electrophoresed on 2% agarose gels containing ethidium bromide and photographed under UV illumination. cDNA fragments were extracted from agarose gels, purified using a agarose gel purification kit (Axygen, Union City, CA, USA), and cloned to pEASY-T1 simple vector (TransGen, Beijing, China). The cloned product was sequenced using automated DNA sequencer (Applied Biosystems 3730, Shanghai, China).

### 3.5. Phylogenetic Analysis

In order to construct the phylogenetic tree for CDA genes, the protein sequence of the candidate gene was used as the query sequence to search for the homologous sequences of silkworm in SilkDB [42,43] or other species in NCBI. Using amino acid sequences of the CDA conserved core domain, the phylogenetic relationship of insect CDAs was reconstructed by Neighbor-Joining (NJ) methods in program MEGA5 [44]. The evolutionary distance was estimated by implementing the JTT amino acid matrix. The pairwise deletion option was used in the NJ tree reconstruction and the accuracy of the tree topology was assessed by bootstrap analysis with 1000 resampling replicates.

### 3.6. Expression Profile Analysis of Putative BmCDA7

For temporal expression analysis, three whole silkworm bodies (except for the midgut contents) were collected at the times of the first-fourth instar larvae, middle-stage silkworm and molting silkworm. To examine gene expression in specific tissues, the foregut, midgut, hindgut and remaining carcasses were dissected from larvae on day three of the fifth instar. cDNA was synthesized as described above. RT-PCRs were done using the following program: initial incubation at 94 °C for 4 min, followed by 25 cycles at 94 °C for 40 s, 40 s of annealing, 1 min extension at 72 °C and a final extension at step at 72 °C for 10 min. PCR products were separated by electrophoresis in 1.5% (*w*/*v*) agarose gels and stained with ethidium bromide. The primer sequences used in this part of the study are shown in Supplementary Information Table S4.

### 3.7. Prokaryotic Expression, Preparation of Antiserum and Immunobloting

The sequence of cDNA encoding *BmCDA7* was amplified by PCR. The forward and reverse primers each contained one restriction enzyme site (*NdeI* and *XhoI*, respectively), underlined in Table S4. The PCR product was cloned into the pEASY-T1 simple vector (TransGen, Beijing, China) for DNA sequencing. The *BmCDA7* gene excised from the pEASY-T1 simple vector by *NdeI* and *XhoI* was subcloned into pET-28a. The recombinant plasmid, named pET-28a-BmCDA7, was transformed into *E. coli* strain BL21 (DE3). Expression of the recombinant protein BmCDA7 were induced by 1 mM IPTG for 4 h at 37 °C and confirmed by SDS-PAGE, then purified by incubating the supernatant with Ni-NTA Super-flow beads (Qiagen, Valencia, CA, USA) according to the manufacturer’s instructions.

Polyclonal antibodies against BmCDA7 were produced according to the traditional method. A 1 mg sample of purified recombinant proteins was used to immunize one New Zealand rabbit. The proteins in 1 mL of phosphate-buffered saline and mixed with complete Freund’s adjuvant (1:1, *v*/*v*) were injected into the rabbit on day zero of the first immunization and immunizations were boosted with incomplete Freund’s adjuvant (1:1, *v*/*v*) on days 10, 20 and 30. The antiserum was collected on day 40 and purified with Protein G.

As described above, proteins for use in western blotting were extracted from the foregut, midgut, hindgut and remaining carcasses, which were dissected from larvae on day three of the fifth instar. The protein samples mixed with SDS-PAGE sample buffer were heated in boiling water for 10 min and loaded onto an SDS-PAGE (15% (*w*/*v*) polyacrylamide gel. After electrophoresis, the proteins were blotted onto a polyvinylidene difluoride (PVDF) membrane at a constant current of 200 mA at 4 °C for 50 min. The membrane was incubated in 5% (*v*/*v*) fat free milk in Tris-buffered saline, pH 8.0, 0.1% (*v*/*v*) Tween-20) at 4 °C overnight. The target proteins were detected by probing the blot with a primary antibody (anti-BmCDA7 antiserum, 1:20.000) followed by a secondary goat anti-rabbit antibody conjugated to horseradish peroxidase (1:40.000). The bands were detected by ECL advance Western Blotting Detection Reagents (GE Healthcare, Milwaukee, MI, USA). The tubulin protein was used as the positive control in this analysis and the tubulin antibody was purchased from Sigma.

### 3.8. Eukaryotic Expression and Enzyme Assay

Because the recombinant BmCDA7 protein produced in the prokaryotic expression system showed no enzyme activity (data not shown), the eukaryotic expression system was used to detect the function of the BmCDA7 protein. The method was as described [45]. The cDNA sequences of *BmCDA7* were produced by PCR with a specific primer, which contained an *EcoRI* site and a *NotI* site, (underlined) in Table S4. The purified PCR product was cloned into the pEASY-T1 simple vector (TransGen, Beijing, China) for DNA sequencing. The *BmCDA7* gene from the pEASY-T1-BmCDA7 was ligated between the *EcoRI* and *NotI* sites of the pPIC9k vector (Invitrogen, Carlsbad, CA, USA) using T4 DNA ligase (TaKaRa, Dalian, China). Plasmid DNA (pPIC9k-BmCDA7) was isolated and linearized with *PmeI* and transformed into *P. pastoris* (GS115) cells by electroporation. The screening of positive clones (pPIC9k-BmCDA7) followed the manufacturer’s (Invitrogen, Carlsbad, CA, USA) recommendations. A pPIC9k-BmCDA7 colony was inoculated into 25 mL of Buffered Glycerol-complex Medium (BMGY) grown at 30 °C in a shaker incubator until the absorbance at 600 nm (*A*_600_) reached 2–6. The cells were harvested by centrifugation and suspended to an *A*_600_ value of 1 in 100 mL of BMMY medium and cultured continuously at 30 °C for 96 h. Expression of the BmCDA7 gene was induced by 1% (final concentration) methanol every 24 h. The yeast cells containing empty pPIC9K vector were used as the negative control. The BMMY medium was centrifuged at 12,000*g* for 30 min at 4 °C to remove yeast cells. The proteins in the supernatant were precipitated by 80% saturated ammonium sulfate (final concentration) overnight at 4 °C and then centrifuged at 12,000*g* for 30 min at 4 °C. The pellets were suspended in 3 mL of 50 mM sodium phosphate buffer (pH 7.4).

Li [46] developed a procedure for measuring CDA activity assay based on Srinivasan’s patent [47], in which the CDA catalyzes the conversion of *p*-nitroacetanilide to *p*-nitroaniline (Figure S2). This method was used in our study to measure the CDA activity of BmCDA7. In order to detect the activity of putative BmCDA7, the tests were done in a 10 mL centrifuge tube in a total volume of 5 mL. First, 3 mL of 50 mM sodium phosphate buffer (pH 7.4) was incubated for 15 min at 50 °C, then 1 mL of 200 mg/L aqueous solution of *p*-nitroacetanilide and 1 mL of crude recombinant enzyme solution were added and mixed. The reaction was incubated at 50 °C for 15 min in a water bath. After incubation, the reaction was quenched in a boiling water bath and proteins were removed by low-speed centrifugation for 10 min. The supernatant was analyzed by measuring the absorbance at *A*_400_ in a spectrophotometer. The inactivated crude recombinant enzyme solution was used as the control. The enzymatic activity (*EA*) was computed as:

(1) $$EA=(A_{400}-A_{0})/KT$$

where *EA* is the enzymic activity of the crude recombinant enzyme solution, *A*_400_ is the activity of the crude recombinant enzyme solution, *A*_0_ is the activity of the crude recombinant enzyme solution that was inactivated, *K* is the linear coefficient (0.0807) and *T* is the enzymatic reaction time. One unit of activity was defined as the amount of enzyme required to produce 1 μg of *p*-nitroaniline per hour.

## 4. Conclusions

To date, few studies have focused on the molecular structure and function of the PM, and how the chitin fibrils and proteins assemble to form the PM is unclear. Here, we describe the isolation and characterization of a cDNA encoding a silkworm CDA-like PM protein. This study showed BmCDA7 is one of the PM proteins and has CDA activity, but is different from the peritrophin-type chitin-binding proteins. The functions and regulatory mechanism of BmCDA7 warrant further investigation.

## Figures and Tables

**Table 1.** Characterization of the major PM Proteins of silkworm larvae.

Spot	Protein Name	Cell Function	NCBI Entry	SilkDB Entry	*M*_r_ (kDa)	pI	Seq. (%)
1	H+ transporting ATP synthase beta subunit isoform 1	Proton pump, Proton transport	NP_001040450.1	BGIBMGA012549	54.8	5.1	41.3
2	H+ transporting ATP synthase beta subunit isoform 2	Proton pump, Proton transport	NP_001041705.1	BGIBMGA012555	55.0	5.2	23.8
4	Chitin deacetylase	Enzymatic deacetylation of chitin	-	BGIBMGA013757	41.4	5.2	
5	Unknown						
6	Actin3	Cytoskeleton	P04829	BGIBMGA013945	41.9	5.3	50.0
7	Chaperonin	Protein folding	NP_001073348	BGIBMGA011508	59.2	5.3	24.2
8–10	Unknown						
11	Signal sequence receptor beta subunit	Translocon-associated protein	NP_001040332	BGIBMGA005769	16.4	6.9	
12	Translationally controlled tumor protein	Translation factor activity	NP_001037572.1	BGIBMGA003073	19.6	4.5	30.2
13	Juvenile hormone binding protein	Carring hormone	-	BGIBMGA010979	23.5	5.2	
14	Unknown						
15	Chlorophyllide A binding protein precursor	Candidate receptor proteins of Bt	NP_001037071	BGIBMGA004806	315.5	5.1	
16	ADP-ribosylation factor-like protein	Unknown	NP_001040168	BGIBMGA010943	21.3	6.5	39.0
17	Peritrophic membrane chitin binding protein 2	Chitin binding protein	-	BGIBMGA001491	15.2	7.5	20.4
18–24	Unknown						
25–30	Triacylglycerol lipase	Lipid metabolism	NP_001040159	BGIBMGA010400	31.8	8.8	30.8

**Table 2.** The activity of recombinant BmCDA7 protein produced in *P. pastoris*.

Absorbance		Activity
*A*_400_	*A*_0_	*EA*
0.36	0.21	1.85
